# Comparing the Effect of Aromatherapy with Peppermint and Lavender Essential Oils on Fatigue of Cardiac Patients: A Randomized Controlled Trial

**DOI:** 10.1155/2021/9925945

**Published:** 2021-09-14

**Authors:** Somayeh Mahdavikian, Masoud Fallahi, Alireza Khatony

**Affiliations:** ^1^Nursing Department, Nursing and Midwifery School, Kermanshah University of Medical Sciences, Kermanshah, Iran; ^**2**^ Clinical Research Development Center of Imam Reza Hospital, Kermanshah University of Medical Sciences, Kermanshah, Iran; ^**3**^ Social Development and Health Promotion Research Center, Health Institute, Kermanshah University of Medical Sciences, Kermanshah, Iran; ^4^Infectious Diseases Research Center, Kermanshah University of Medical Sciences, Kermanshah, Iran

## Abstract

**Methods:**

This randomized controlled clinical trial was conducted on 105 cardiac patients. They were randomly divided into three groups: peppermint essential oil (*n* = 35), lavender essential oil (*n* = 35), and control (*n* = 35). Fatigue Severity Scale (FSS) was used to collect data. The intervention was performed for 7 nights. Before and after the intervention, the questionnaire was completed by all patients. In each intervention group, patients inhaled 3 drops of lavender or peppermint essential oils. In the control group, patients inhaled 3 drops of aromatic placebo.

**Results:**

The results showed the average fatigue decreased in the study groups. There was no statistically significant difference between the two groups of lavender and peppermint in terms of mean fatigue after the intervention. However, there was a statistically significant difference between lavender and control groups (*P* < 0.001), as well as peppermint and control groups (*P* < 0.001).

**Conclusion:**

Aromatherapy with peppermint and lavender essential oils can reduce the fatigue of cardiac patients, so the use of these fragrances is recommended.

## 1. Introduction

Fatigue is one of the most common chronic and annoying complaints in cardiac patients [1]. Fatigue causes respiratory problems, insomnia, depression, and isolation [1, 2]. Further, chronic fatigue in patients may lead to suicide [3]. Various studies have reported the prevalence of fatigue in cardiac patients to be more than 50% [1, 2, 4].

There are several treatments to reduce fatigue, including diet, medication, psychotherapy, and herbal remedies. Although the existing drugs reduce or eliminate the patients' fatigue [5–8], they are associated with problems, such as drug interactions and side effects, or have no absolute effect on fatigue relief [9, 10]. Therefore, it is necessary to use relatively safer methods with fewer side effects.

Today, nonpharmacological methods, such as aromatherapy, are used to reduce fatigue [11]. Aromatherapy is a natural method that has been used for a long time to relax the body and soul and to treat some diseases [12, 13]. Peppermint and lavender are among the most important aromas used in aromatherapy [14]. Peppermint (*Mentha × piperita*) belongs to the family Lamiaceae [14, 15] and has been used as a rubefacient for centuries. This plant reduces heart rate and systolic blood pressure, relaxes bronchial smooth muscles, and increases ventilation [16]. The medicinal properties of peppermint are due to the presence of menthol, which is the main ingredient in peppermint essential oil. The main effects of peppermint include analgesic, anxiolytic, sedative, and sleep quality enhancer [14, 15]. Lavender belongs to the genus *Lavandula*, which is scientifically known as *Lavandula angustifolia* Mill. and belongs to the Lamiaceae family [14, 17, 18]. In traditional medicine, lavender is used as an analgesic, for massage therapy as well as inhalation therapy [19]. Regarding the effect of lavender on fatigue in cardiac patients, there is very limited evidence that shows the effectiveness of this essential oil [20, 21], but at the same time, more studies are needed [20, 22]. Among the therapeutic effects of lavender, which have been mentioned in some studies, are sleep quality enhancer, antistress, and sedative [14, 17, 18]. Limited studies have been performed on the effect of aromatherapy on patients' fatigue levels [23, 24]. In this regard, the results of a study indicate the effect of lavender on reducing fatigue in cardiac patients [22]. Lavender has shown different results in hemodialysis and hospitalized patients [19, 25, 26].

Aromatherapy with peppermint essential oil has been used to reduce fatigue in hypothyroid and prediabetic patients and has been associated with positive results [27, 28], but no study has been performed on cardiac patients so far. Due to the limited number of studies on the effect of inhalation aromatherapy with lavender and peppermint essential oils on fatigue in cardiac patients as well as their different results, the present study was conducted to compare the effect of lavender and peppermint essential oils on fatigue in cardiac patients. This study sought to answer the following questions:

1. What is the level of fatigue before the intervention in the study groups?
2. What is the level of fatigue after the intervention in the study groups?

The research hypothesis was that inhalation aromatherapy with essential oils of lavender and peppermint could reduce fatigue in cardiac patients.

## 2. Materials and Methods

### 2.1. Trial Design

This randomized controlled clinical trial was conducted with a parallel design and 1 : 1 allocation ratio in the experimental (including two groups of aromatherapy) and control groups. This study was based on the CONSORT guidelines. It lasted for 1 year, from January 2019 to January 2020.

### 2.2. Sample and Sampling Method

The study population included all cardiac patients admitted to the coronary care unit (CCU) of Imam Ali Hospital. This hospital is the largest cardiology center in western Iran, Kermanshah. The sample size was determined to be 26 patients in each group using the results of the study of Bagheri-Nesami et al. (2016) [19], with 95% confidence and 80% power. In order to achieve more reliable results and increase the test power, the sample was increased to 35 patients in each group, so a total of 105 patients were included in the study.

The samples were included in the study by convenience sampling method and were randomly divided into aromatherapy with lavender and peppermint groups and control group. To perform blocked randomization, aromatherapy with peppermint and lavender groups and control group was assigned codes A, B, and C, respectively. The blocks included ABC, ACB, BAC, BCA, CAB, and CBA. The first block was selected by lot, which was ABC block. The third author generated the random list, and the second author recruited the samples into the groups.

The inclusion criteria consisted of patient and physician satisfaction, having passed at least 48 hours of hospitalization, full consciousness, absence of respiratory problems, Fatigue Severity Scale (FSS) score >36, stability of vital signs (blood pressure, pulse, respiration, and temperature), age range of 18–65 years, absence of smoking and drug use, not drinking caffeinated beverages one hour before the intervention (because of the close relationship between insomnia and fatigue (29)), no history of any allergies based on patient statements, and a healthy sense of smell. In order to evaluate the health of the sense of smell, the openness of the nostrils was examined by the third author, who is a professor of nursing. In addition, a cardamom seed was placed separately in front of each nostril and if diagnosed, was a sign of olfactory health.

The exclusion criteria included patient's death or discharge and administration of narcotics or oxygen during aromatherapy.

### 2.3. Measurement Instruments

The study instruments included a demographic questionnaire and FSS. The demographic questionnaire included questions on age, gender, education, occupation, marital status, type of heart disease, and history of myocardial infarction. FSS is a standard tool developed by Lauren B. Krupp et al. (1988) [29]. The validity and reliability of this tool have been investigated in previous studies [19, 30]. Azimian et al. (2009) examined the internal consistency of FSS using Cronbach's alpha and reported an alpha of 0.96 [19]. The Persian version of FSS has also been psychometrically evaluated in Iran by Fereshteh Nejad et al. (2013). In their study, the internal consistency method was used to evaluate the reliability and Cronbach's alpha was 0.96, indicating an acceptable index [30]. In the present study, the internal consistency of FSS was examined by Cronbach's alpha method, and the alpha coefficient was 0.89.

The FSS consists of 9 seven-point Likert scale questions, with responses ranging from 1 (strongly disagree) to 7 (strongly agree). The score range of the questionnaire is between 9 and 63. A score ≤40 indicates mild fatigue, and >40 shows severe fatigue [30, 31].

### 2.4. Interventions

After obtaining the approval from the University Ethics Committee, the researcher (first author) referred to Imam Ali Hospital for sampling. This hospital is the most advanced cardiovascular center in western Iran, and its CCU_1_ has 15 single beds separated by glass walls and curtains. At first, the objectives of the study were explained to the samples, and if they were willing, they were included in the study. In the intervention groups, inhalation aromatherapy was performed with 100% pure scents of lavender and peppermint essential oils. These scents were produced by Zardband Pharmaceutical Company (Yasuj, Iran). In the control group, the same amount of aromatic placebo was used. For this purpose, a very small amount of lavender essential oil (as plenty as micrograms) was added to the propylene glycol solution to create a sense of aromatic substances [32].

**Figure 1:**
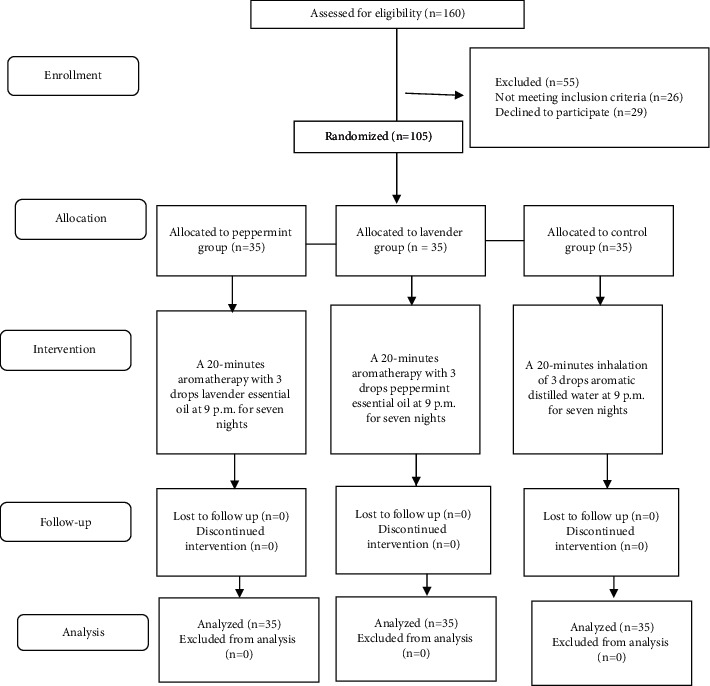
The CONSORT diagram of the study.

The intervention took 7 nights. In the intervention groups, at 9 : 00 pm, 3 drops of each fragrance were sprayed on a cotton ball and attached to the patient's collar for 20 minutes. The same procedure was performed for the control group with 3 drops of aromatic distilled water. On the first night before the start of the study, the demographic questionnaire and FSS were completed by all subjects. It should be noted that due to the specific odor of each fragrance, blinding was not performed in this study. The researcher did his best to minimize environmental stimuli, such as light and noise. The study process is shown in Figure 1.

### 2.5. Essential Oils

Lavender and peppermint essential oils were obtained from Zardband Pharmaceuticals Company (Yasouj, Iran). The essential oils were stored in a dark place at 2–8°C. Moreover, the expiration date of these essential oils was carefully examined by the researcher, and they were consumed before the expiration date. Analysis of the composition of lavender and peppermint extracts is mentioned in the supplementary section (Supplementary materials).

### 2.6. Statistical Methods

Data were analyzed by Statistical Package for the Social Sciences (SPSS v.18.0; SPSS Inc., Chicago, IL, USA). Kolmogorov–Smirnov test was used to determine the distribution of the fatigue variable, and results showed the abnormal distribution of the variable. Chi-square test was also used to determine whether the groups were homogeneous in terms of nominal variables, including gender, marital status, occupation, education, type of cardiac disease, history of diabetes, myocardial infarction, and aromatherapy. To compare the fatigue score among the study groups, Kruskal–Wallis H test was used in each stage before and after the intervention. Wilcoxon signed-rank test was also used to compare fatigue scores before and after the intervention in each study group. Mann–Whitney *U* test was used for pair comparison of fatigue score in the study groups. The level of significance for all tests was less than 0.05.

### 2.7. Ethical Considerations

This trial was conducted in accordance with the Declaration of Helsinki. The Ethics Committee of Kermanshah University of Medical Sciences approved the study protocol with reference number IR.KUMS.REC.1397.195. The study was also registered at the Iranian Registry of Clinical Trials under the code IRCT20181105041563N2. Before the study, the objectives and methods were explained to all of the participants, and they were assured that their responses would remain confidential. Written informed consent was obtained from all participants before the study. Permission was also obtained from the physicians of the patients participating in the study.

## 3. Results

In this study, 105 patients were divided into two intervention groups and one control group (35 patients in each group). The mean age of the samples was 54.0 ± 8.1 years. Most of the patients were male (*n* = 84, 80%), were married (*n* = 85, 81.8%), and had undergraduate education (*n* = 81, 77.1%). About half of the patients were retired (*n* = 57, 54.3%). Further, 40.0% (*n* = 42) of patients had a history of myocardial infarction, and 54.3% of patients (*n* = 57) were diagnosed with coronary artery disease. All three peppermint, lavender, and control groups were homogeneous in terms of all demographic variables, including age, sex, marital status, body mass index, occupation, education, type of cardiac disease, and history of aromatherapy, myocardial infarction, and diabetes (Table 1).

Fatigue scores in the peppermint group before and after the intervention were 49.7 ± 3.8 and 25.4 ± 5.3 out of 63, respectively, which showed a statistically significant difference (*P* < 0.001). In the lavender group, the fatigue scores before and after the intervention were 46.3 ± 4.3 and 25.2 ± 8.9, respectively, which also indicated a statistically significant difference (*P* < 0.001). Moreover, fatigue scores in the control group before and after the intervention were 47.5 ± 4.5 and 45.6 ± 9.9, respectively, which also showed a statistically significant difference (*P*=0.046). Before the study, there was no statistically significant difference between the study groups in terms of fatigue, but this difference was statistically significant after the intervention (*P* < 0.001) (Table 2).

There was no statistically significant difference between the two groups of lavender and peppermint in terms of mean fatigue score after the intervention, but there was a significant difference between the lavender and control groups (*P* < 0.001) and peppermint and control groups (*P* < 0.001 (Table 3)).

## 4. Discussion

The aim of this clinical trial was to compare the effect of inhalation aromatherapy with lavender and peppermint essential oils on the fatigue level of cardiac patients. The results of the study showed the positive and identical effect of both lavender and peppermint essential oils on reduction of mean fatigue in cardiac patients. Various studies have investigated the effect of aromatherapy with lavender and peppermint essential oils on the level of fatigue [26–28]. Another study investigated the effect of aromatherapy with lavender essential oil on the fatigue level of 50 elderly people, and results showed the significant effectiveness of this fragrance in reducing fatigue [13]. Inhalation aromatherapy with lavender essential oil may reduce patients' fatigue by improving sleep quality, increasing relaxation, and reducing anxiety [33–36]. In this regard, some studies have investigated the effect of lavender essential oil on sleep quality in patients with coronary artery disease and the elderly living in a nursing home and have indicated the effectiveness of this aroma in improving sleep quality [34, 35]. In a clinical trial, the effect of inhalation aromatherapy with lavender essential oil on fatigue of hemodialysis patients was investigated. The intervention group (*n* = 29) received inhalation aromatherapy with 5% lavender essential oil 3 times during hemodialysis. The control group (*n* = 30) received only routine care. The duration of the study was four weeks. The results showed that aromatherapy with lavender essential oil had no effect on patients' fatigue [19]. The main limitation of this study is that patients were not matched in terms of fatigue at the time of enrollment. But in the current study, only patients whose mean fatigue from the FSS questionnaire was more than 36 were included. In another study, the effect of inhalation aromatherapy with extracts of lavender (*n* = 15) and sweet orange (*n* = 15) on fatigue of hemodialysis patients was investigated. Each of the two intervention groups included 15 patients. Aromatherapy was performed three times a week for two consecutive weeks. In each group, a drop of the extract was poured on a piece of gauze and attached to the patient's collar overnight after dialysis. Patients' fatigue was assessed at the end of each week. The results showed that aromatherapy with lavender had no effect on fatigue, but aromatherapy with orange extract was effective [37]. Lack of control group and small sample size are the limitations of this study. In our study, the sample size was sufficient and there was a control group.

In the present study, the mean fatigue after the intervention in the peppermint group was significantly reduced. There has been no study on the effect of aromatherapy with peppermint essential oil on the level of fatigue in cardiac patients, and existing studies have also been performed on noncardiac patients, whose results are contradictory [27, 28, 38].

Safajou et al. investigated the effect of inhaled aromatherapy with a combination of lemon and peppermint essential oils on nausea, vomiting, and fatigue among pregnant women. The results showed that aromatherapy reduced nausea and vomiting but had no effect on fatigue [32]. Blackburn et al. evaluated the effect of inhalation aromatherapy with three essential oils of mint, lavender, and chamomile on the fatigue levels of 50 patients with leukemia. The results showed the positive effect of aromatherapy on fatigue reduction [39]. Blackburn et al. did not assess the effects of peppermint, lavender, and chamomile essential oils separately, while the present study investigated the effects of lavender and peppermint essential oils separately. Peppermint essential oil contains menthol and menthol compounds that affect the olfactory pathways of the brain, reduce anxiety, relieve pain, increase relaxation, reduce pulse rate, and increase sleep quality [14, 15, 38]. These effects can play a role in reducing the level of fatigue.

The results showed that the mean fatigue decreased in the control group, but the level of reduction was very small compared to the lavender and peppermint groups. This finding is consistent with the results of previous studies [14, 15]. This decrease might be due to similar environmental effects in all three groups and the psychological and self-inductive effects of using aromatic distilled water in the control group.

In the present study, patients in the intervention and control groups were homogeneous in terms of demographic variables, so the study was not threatened by the cofounding variables. Although this intervention was performed on hospitalized patients who were in the same state of physical activity, other factors affecting fatigue, such as psychological factors or medications, were not controlled.

This study faced several limitations. Factors such as the severity of heart disease, psychological factors, and medications might be associated with the patients' fatigue, which were not studied in this research. Another limitation was the lack of gas chromatography-mass spectrometry due to financial constraints, but Zarband Pharmaceuticals Company, which produces essential oils, is approved by the Ministry of Health of Iran and is an ISO 9001 : 2008-certified company.

## 5. Conclusion

Inhalation aromatherapy with lavender and peppermint essential oils can reduce the level of fatigue in cardiac patients. Due to the effectiveness of these fragrances, they are recommended to be used to reduce the fatigue of patients admitted to the CCU. It is suggested that future studies investigate the effect of different concentrations of lavender and peppermint essential oils on the level of fatigue in cardiac patients.

## Figures and Tables

**Table 1:** Comparison of the demographic variables in the study groups.

Variables		Groups			*P* value^d^
		Peppermint *N* (%)	Lavender *N* (%)	Control *N* (%)	
Age (years)	33–43	5 (14.3)	3 (8.6)	4 (11.4)	0.629
	44–54	8 (22.9)	14 (40.0)	12 (34.3)	
	55–65	22 (62.9)	18 (51.4)	19 (54.3)	

Gender	Female	9 (25.7)	7 (20.0)	5 (14.3)	0.490
	Male	26 (74.3)	28 (80.0)	30 (85.7)	

Marital status	Single	5 (14.3)	8 (22.9)	7 (20.0)	0.490
	Married	30 (85.7)	27 (77.1)	28 (80.0)	

BMI (kg/m^2^)	≥18.5	0 (0)	0 (0)	0 (0)	0.434
	18.51–24.99	5 (14.3)	9 (25.7)	8 (22.9)	
	25–29.99	18 (51.4)	20 (57.1)	20 (57.1)	
	30≤	12 (34.3)	6 (17.1)	7 (20.0)	

Job	Self-employment	10 (28.6)	8 (22.9)	11 (31.4)	0.744
	Retired	18 (51.4)	19 (54.3)	20 (57.1)	
	Housekeeper	7 (20.0)	8 (22.9)	4 (11.4)	

Education status	Nonacademic	27 (77.1)	26 (74.3)	28 (80.0)	0.850
	Academic	8 (22.9)	9 (25.7)	7 (20.0)	

Place of residence	City	22 (62.8)	24 (68.6)	26 (74.3)	0.588
	Village	13 (37.1)	11 (31.4)	9 (25.7)	

Type of cardiac disease	MI^†^	9 (25.7)	7 (20.0)	10 (28.6)	0.865
	CAD^‡^	20 (57.1)	19 (54.3)	18 (51.4)	
	CHF^§^	6 (17.1)	9 (25.7)	7 (20.0)	

Diabetes history	Yes	17 (48.6)	14 (40.0)	16 (45.7)	0.764
	No	18 (51.4)	21 (60.0)	19 (54.3)	

MI history	Yes	16 (45.7)	14 (40.0)	12 (34.3)	0.621
	No	19 (54.3)	21 (60.0)	23 (65.7)	

Inhalation aromatherapy history	Yes	14 (40.0)	10 (28.6)	12 (34.3)	0.602
	No	21 (60.0)	25 (71.4)	23 (65.7)	

^†^Myocardial infarction; ^‡^coronary artery diseases; ^§^congestive heart failure; ^d^based on chi-square test.

**Table 2:** Comparison of the fatigue levels before and after the intervention in aromatherapy and control groups.

	Study groups									*P* value ^$^
	Peppermint			Lavender			Control			
Intervention	Mean ± SD^•^	Med (IQR^#^)	95% ci^‡^	Mean ± SD	Med (IQR)	95%ci	Mean ± SD	Med (IQR)	95% ci	
Before	49.7 ± 3.8	51 (3)	48.4, 51.0	46.3 ± 4.3	47| [5]	44.8, 47.8	47.5 ± 4.5	49 (6)	45.8, 49.1	0.896
After	25.4 ± 5.3	24 (3)	23.6, 27.2	25.2 ± 8.9	23 (5)	22.1, 28.3	45.6 ± 9.9	49 (9)	42.2, 49.1	<0.001
*P* value	<0.001^†^			<0.001^†^			0.046^†^			

^•^Standard deviation; ^‡^confidence interval; ^$^based on Kruskal–Wallis H test; ^#^interquartile range; ^†^Wilcoxon signed-rank test.

**Table 3:** Comparing the study groups in terms of the fatigue levels after intervention.

Groups	Median (IQR^$^)	95% ci	Effect size ^ǁ^	*P* value^†^	Z
Peppermint	24 (3)	23.6, 27.2	0.2	0.645	–0.461
Lavender	23 (5)	22.1, 28.3			

Peppermint	24 (3)	23.6, 27.2	–20.2	<0.001	–6.235
Control	49 (9)	42.2, 49.1			

Lavender	23 (5)	22.1, 28.3	–20.4	<0.001	–5.972
Control	49 (9)	42.2, 49.1			

^$^Interquartile range; ^†^based on Mann–Whitney *U* test; ^ǁ^calculated based on the difference of means.

## Data Availability

The identified datasets analyzed during the current study are available from the corresponding author on reasonable request.

## Supplementary Materials

Supplementary Materials 1. Analysis of lavender extract compounds. 2. Analysis of peppermint extract compounds. 3. FSS questionnaire.
