# Supplementary material for: Comparing the Effect of Aromatherapy with Peppermint and Lavender Essential Oils on Fatigue of Cardiac Patients: A Randomized Controlled Trial
Source: Evid Based Complement Alternat Med. 2021 Sep 14;2021:9925945. doi: 10.1155/2021/9925945 (PMC8457936; doi:10.1155/2021/9925945)
Supplement: Supplementary Materials — 1. Analysis of lavender extract compounds. 2. Analysis of peppermint extract compounds. 3. FSS questionnaire. [file 9925945.f1.zip › 9925945.f1/FSS Questionaire (1).pdf]

Please circle the number between 1 and 7 which you feel best fits the following statements. This refers to your usual way of life within the last week. 1 indicates "strongly disagree" and 7 indicates "strongly agree."

Read and circle a number. Strongly Disagree → Strongly Agree 1.

1. My motivation is lower when I am fatigued. 1 2 3 4 5 6 7 2.
2. Exercise brings on my fatigue. 1 2 3 4 5 6 7 3.
3. I am easily fatigued. 1 2 3 4 5 6 7 4.
4. Fatigue interferes with my physical functioning. 1 2 3 4 5 6 7 5.
5. Fatigue causes frequent problems for me. 1 2 3 4 5 6 7 6.
6. My fatigue prevents sustained physical functioning. 1 2 3 4 5 6 7 7.
7. Fatigue interferes with carrying out certain duties and responsibilities. 1 2 3 4 5 6 7 8.
8. Fatigue is among my most disabling symptoms. 1 2 3 4 5 6 7 9.
9. Fatigue interferes with my work, family, or social life. 1 2 3 4 5 6 7
