# Supplementary material for: Comparing the Effect of Aromatherapy with Peppermint and Lavender Essential Oils on Fatigue of Cardiac Patients: A Randomized Controlled Trial
Source: Evid Based Complement Alternat Med. 2021 Sep 14;2021:9925945. doi: 10.1155/2021/9925945 (PMC8457936; doi:10.1155/2021/9925945)
Supplement: Supplementary Materials — 1. Analysis of lavender extract compounds. 2. Analysis of peppermint extract compounds. 3. FSS questionnaire. [file 9925945.f1.zip › 9925945.f1/Supplementary description_9925945.docx]

**Supplementary materials**

1. The analysis result of Lavender essential oil

2. The analysis result of Peppermint essential oil

3. FSS Questionaire
